# Marine Biotoxins: Occurrence, Toxicity, Regulatory Limits and Reference Methods

**DOI:** 10.3389/fmicb.2016.01051

**Published:** 2016-07-06

**Authors:** Pierina Visciano, Maria Schirone, Miriam Berti, Anna Milandri, Rosanna Tofalo, Giovanna Suzzi

**Affiliations:** ^1^Faculty of Bioscience and Technology for Food, Agriculture and Environment, University of TeramoTeramo, Italy; ^2^Istituto Zooprofilattico Sperimentale dell’Abruzzo e del Molise “G. Caporale”Teramo, Italy; ^3^National Reference Laboratory for Marine Biotoxins, Fondazione Centro Ricerche MarineCesenatico, Italy

**Keywords:** shellfish poisoning, toxicity, symptoms, human health, detection method

## Abstract

Harmful algal blooms are natural phenomena caused by the massive growth of phytoplankton that may contain highly toxic chemicals, the so-called marine biotoxins causing illness and even death to both aquatic organisms and humans. Their occurrence has been increased in frequency and severity, suggesting a worldwide public health risk. Marine biotoxins can accumulate in bivalve molluscs and regulatory limits have been set for some classes according to European Union legislation. These compounds can be distinguished in water- and fat-soluble molecules. The first group involves those of Paralytic Shellfish Poisoning and Amnesic Shellfish Poisoning, whereas the toxins soluble in fat can cause Diarrheic Shellfish Poisoning and Neurotoxic Shellfish Poisoning. Due to the lack of long-term toxicity studies, establishing tolerable daily intakes for any of these marine biotoxins was not possible, but an acute reference dose can be considered more appropriate, because these molecules show an acute toxicity. Dietary exposure assessment is linked both to the levels of marine biotoxins present in bivalve molluscs and the portion that could be eaten by consumers. Symptoms may vary from a severe gastrointestinal intoxication with diarrhea, nausea, vomiting, and abdominal cramps to neurological disorders such as ataxia, dizziness, partial paralysis, and respiratory distress. The official method for the detection of marine biotoxins is the mouse bioassay (MBA) showing some limits due to ethical restrictions and insufficient specificity. For this reason, the liquid chromatography–mass spectrometry method has replaced MBA as the reference technique. However, the monitoring of algal blooms producing marine biotoxins should be regularly assessed in order to obtain more reliable, accurate estimates of bloom toxicity and their potential impacts.

## Introduction

**FIGURE 1:**
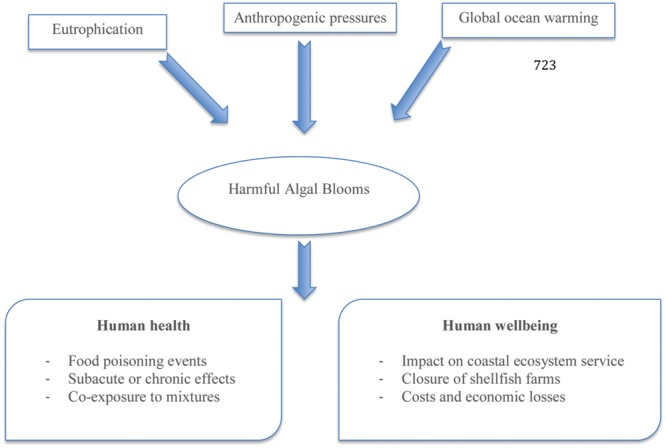
**Factors involved in the occurrence of harmful algal blooms and the consequences on human health and wellbeing (modified from Berdalet et al., 2015)**.

Harmful algal blooms (HAB) are natural phenomena carried out by the overgrowth of marine phytoplankton (Ferreiro et al., 2015). Over the last decades the occurrence and intensity of HAB appear to be increasing on a global scale due to rising ocean temperatures and growing coastal eutrophication (McCarthy et al., 2015). The geographical expansion of HAB can also be associated with ballast waters transporting encysted algae to new environments or massive algae spreading caused by aquaculture practices (Anderson et al., 2002; Maso and Garcés, 2006; Smayda, 2007). Among the thousands of microalgal species known in nature, about 300 are involved in harmful events and more than 100 (of these species) produce persistent natural toxins that can cause intoxication or even death in humans and animals. Moreover, such toxic outbreaks can have consequences on other components of human wellbeing both in terms of socio-economic impact and costs. The main factors affecting HAB occurrence and their influence on the environment, shellfisheries and consumers are shown in **Figure 1**. The direct impact of HAB on human health is linked to poisoning after consumption of contaminated seafood, skin contact with contaminated water, and/or inhalation of aerosolized biotoxins. Nowadays the increasing of information about human exposure hazards as well as the strategies able to prevent HAB occurrence in seafood need to be understood and improved, because such phenomenon results from complex interactions among physical, chemical, and biological processes in the marine environment (Berdalet et al., 2015). Moreover, even if the classic description of intoxication due to marine biotoxins is represented by acute symptoms, there is also little information about the impacts of long-term low-level exposure together with epidemiological studies regarding the populations at risk (Lefebvre and Robertson, 2010). The toxicological mechanisms of some marine biotoxins are yet incompletely understood and no observations of adverse effects in humans have been reported.

**FIGURE 2:**
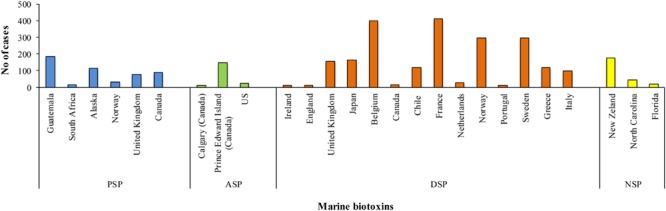
**Outbreaks (number of cases) of poisoning due to marine biotoxins occurred from 1970 through 2010**.

It is well known that marine biotoxins can accumulate in the tissues of some marine organisms, particularly filter-feeding bivalves. In all cases, they are *de novo* produced by certain photo- or mixo-trophic microalgae not by the shellfish, and filter-feeding transfers them to the molluscs (Nielsen et al., 2016). Mussels filter approximately 20 L water/h and during HAB, waters may contain several million algal cells per liter. Some phytoplankton species in these blooms produce phycotoxins that can accumulate through the marine food webs (Ferreiro et al., 2015). Outbreaks of intoxication in humans due to marine biotoxins (**Figure 2**) are caused by the ingestion of contaminated shellfish and can have a wide range of symptoms linked to the specific toxic compound (Richter and Fidler, 2015; Turner and Goya, 2015). Damage to nervous or intestinal system rather than loss of memory have been observed depending on the type of algal bloom. Among them some dinoflagellate and diatom species, such as *Noctiluca scintillans* and *Skeletonema costatum*, are responsible only for discoloration of water and death of fish and other marine organisms, whereas species belonging to the genera *Alexandrium, Gymnodinium, Dinophysis*, and *Pseudo-nitzschia* are the main producers of marine biotoxins for humans (Berti and Milandri, 2014; Bruce et al., 2015).

Marine biotoxins can be distinguished in water- and fat-soluble according to their solubility. On the basis of their poisoning symptoms, they are also classified as toxins causing paralytic shellfish poisoning (PSP), amnesic shellfish poisoning (ASP), diarrheic shellfish poisoning (DSP), neurotoxic shellfish poisoning (NSP), and ciguatera fish poisoning (CFP) (Poletti et al., 2003).

According to their own chemical structure, marine biotoxins are classified into eight groups – namely the azaspiracid (AZA), brevetoxin (BTX), cyclic imine (CI), domoic acid (DA), okadaic acid (OA), pectenotoxin (PTX), saxitoxin (STX), and yessotoxin (YTX) groups. Two additional groups, palytoxin (PlTX) and ciguatoxin (CTX), are also considered (EFSA, 2009a). Marine biotoxins and toxic effects for consumers after the ingestion of contaminated seafood are reported in **Table 1**.

**Table 1:** Classification of marine biotoxins and main adverse effects in humans.

Marine biotoxin	Group	Source	Symptomatology
Saxitoxin	PSP	*Alexandrium* spp. *Gymnodinium catenatum* *Pyrodinium bahamense*	• Gastrointestinal symptoms • Paralytic phenomena • Recovery or death

Domoic acid	ASP	*Pseudo-nitzschia* spp. *Nitzschia* spp.	• Gastrointestinal and neurological symptoms • Cardiac or respiratory problems • Recovery or death

Okadaic acid		*Prorocentrum lima* *Dinophysis* spp.	• Gastrointestinal symptoms • Recovery within 3 days

Pectenotoxin		*Dinophysis* spp.	• Gastrointestinal symptoms

Yessotoxin	DSP	*Protoceratium reticulatum* *Lingulodinium polyedrum* *Gonyaulax spinifera*	• Lack of observations in humans

Azaspiracid		*Amphidoma languida* *Azadinium spinosum*	• Gastrointestinal symptoms

Brevetoxin	NSP	*Karenia brevis*	• Gastrointestinal and neurological symptoms • Respiratory problems • Recovery or death

Ciguatoxin	CFP	*Gambierdiscus* spp.	• Gastrointestinal symptoms • Cardiovascular or neurological problems

Cyclic imine		*Alexandrium* spp. *Karenia* spp.	• Lack of observations in humans

Palytoxin		*Palythoa* spp. *Ostreopsis* spp.	• Gastrointestinal symptoms • Muscle and cutaneous problems

Human exposure to biotoxins generally refers only to occasional consumption and is characterized by acute and short-term events, so that acute reference doses (ARfD) have been established for these toxic compounds rather than tolerable daily intakes that could not be determined due to the lack of appropriate toxicological data. The ARfD are derived from the corresponding concentration of marine biotoxins per kg of shellfish meat when consuming a large portion (400 g) of shellfish. A crucial issue, when deriving both these values from the most relevant toxicological information, is the size of safety factors. Generally, default values of 10 and 100 on the basis of human and animal data are applied, respectively (FAO/IOC/WHO, 2004). Furthermore, the safety factor is usually increased if, due to the critical effect, there is a lowest observable adverse effect level (LOAEL) instead of a no observable adverse effect level (NOAEL). The ARfD, safety factors, LOAEL and NOAEL for each individual marine biotoxin, as well as regulatory limits and maximum levels based on consumption of 400 g of shellfish are reported in **Table 2**.

**Table 2:** Regulatory limits, lowest observable adverse effect level (LOAEL), no observable adverse effect level (NOAEL), and acute reference dose for marine biotoxins (modified by FAO/IOC/WHO, 2004; EFSA, 2009b).

Marine biotoxins	Regulatory limits	Exposure after consumption of 400 g portion at the EU limit	LOAEL (1) NOAEL (2) μg/kg b.w.^∗∗∗^	Safety factors Human data (H) Animal data (A)	Acute reference dose (ARfD)	Maximum concentration in shellfish meat (400 g portion) not exceeding ARfD
Okadaic acid	160 μg OA eq.^∗^/kg SM^∗∗^	64 μg OA eq./kg person	1 (1)	3 (H)	0.3 μg OA eq./kg b.w.	45 μg OA eq./kg SM
Azaspiracid	160 μg AZA eq./kg SM	64 μg AZA1 eq./kg person	0.4 (1)	10 (H)	0.2 μg AZA1 eq./kg b.w.	30 μg AZA1 eq./kg SM
Pectenotoxin	160 μg PTX eq./kg SM	64 μg PTX2 eq./kg person	–	–	0.8 μg PTX2 eq./kg b.w.	120 μg PTX2 eq./kg SM
Yessotoxin	3.75 mg YTX eq./kg SM	400 μg YTX eq./kg person	5000 (2)	100 (A)	25 μg YTX eq./kg b.w.	3.75 mg YTX eq./kg SM
Saxitoxin	800 μg PSP/kg SM	320 μg STX eq./kg person	2 (1)	3 (H)	0.5 μg STX eq./kg b.w.	75 μg STX eq./kg SM
Domoic acid	20 mg DA/kg SM	8 mg DA/kg person	1000 (1)	10 (H)	30 μg DA/kg b.w.	4.5 mg DA/kg SM

*^∗^eq. = equivalent; ^∗∗^SM = shellfish meat; ^∗∗∗^b.w. = body weight; – = not reported.*

The present review focuses on the specific toxicity and epidemiology of marine biotoxins, the regulatory limits set by European Union (EU) legislation for the most toxic compounds as well as the methods of detection and quantification.

## Paralytic Shellfish Poisoning

Paralytic shellfish poisoning, caused by 58 closely related compounds based on a tetrahydropurine skeleton (Burrell et al., 2013), is one of the most studied intoxications with serious symptoms in humans. In particular, it is the result of exposure to saxitoxin (STX) and gonyautoxin (GTX). In 1957, a PSP toxin was isolated in clams (*Saxidomus giganteus*) living in Alaska coastal areas and in 1975 the chemical structure was assigned to STX. The main producers of PSP toxins are dinoflagellates of the genus *Alexandrium* occurring along the Atlantic and Pacific coast (Bernd and Bernd, 2008) but also in the Mediterranean Sea, where other species such as *Gymnodinium catenatum* can be present (Berti and Milandri, 2014).

More than 30 STX analogs have been identified and grouped into four subgroups: carbamate, *N*-sulfo-carbamoyl, decarbamoyl, and hydroxylated saxitoxins (EFSA, 2009a).

The intake of biotoxins necessary to cause human poisoning varies greatly due to the differences in susceptibility among individuals. The symptoms are similar to paralytic phenomena (cramp, signs of paralysis, and blocking of respiration) because PSP toxins are potential neurotoxins blocking the excitation current in nerve and muscle cells (Schirone et al., 2011). Human PSP outbreaks can be distinguished in mild, moderately severe and extremely severe. The symptoms occurring in the mild form include tingling sensation or numbness around the lips, gradually spreading to the face and neck, a prickly sensation in fingertips and toes, headache, dizziness, and nausea. The moderately severe illness is characterized by incoherent speech, progression of prickly sensation to arms and legs, stiffness and non-coordination of limbs, general weakness and feeling of lightness, then slight respiratory difficulty and rapid pulse plus backache as late symptoms. In the extremely severe form muscular paralysis, pronounced respiratory difficulty and a choking sensation may occur. In fatal cases, death is caused by respiratory paralysis occurring within 2–12 h after the consumption of contaminated shellfish, in absence of artificial respiration. Patients who survive PSP for 24 h, with or without mechanical intervention, have a high probability of a full and rapid recovery (FAO/IOC/WHO, 2004).

Blooms of toxic microalgae producing PSP toxins represent an expanding threat to both human health and fishery resources all over the world. In particular, PSP has been recognized for over a century as a clinical entity in the austral part of South America. A study regarding an outbreak in the Patagonia fjords reported that some fishermen were intoxicated by consumption of bivalve *Aulacomya ater* and two of them died after 3–4 h; in that case up to 8575 μg of STX, equivalent/100 g of shellfish meat, were detected by MBA (García et al., 2004).

The highest number of PSP cases (2124 with 120 deaths) was reported in the Philippines from 1983 to 2002 (Ching et al., 2015). The human fatality proportion from PSP varies according to the ability of the local medical system to treat such intoxication. In a short report, the authors referred that 45 people living along the coast of Nicaragua developed symptoms of PSP and a person died. In similar outbreaks in Southeast Asia and Latin America, the case fatality proportion ranged between 2 and 14% whereas no death occurred among more than 200 cases in Europe and North America (Callejas et al., 2015).

## Amnesic Shellfish Poisoning

Domoic acid and other toxic DA isomers, produced mostly by marine diatoms of the genus *Pseudo-nitzschia*, are responsible for ASP. This compound is a cyclic tricarboxylic amino acid with many structural and functional similarities with kainic acid, an analog to glutamic acid. In particular, DA binds with glutamate receptors in central nervous system causing over-stimulation of these receptors with production of reactive oxygen species and sometimes cell death (Schwarz et al., 2014).

In humans, symptoms range from gastrointestinal effects (nausea, vomiting, diarrhea, or abdominal cramps) and/or neurological signs (confusion, lethargy, disorientation, paresthesia, and short-term memory loss) and in extreme cases coma or death.

The accumulation of DA in bivalve tissues depends on several factors, such as the presence of *Pseudo-nitzschia* species and their toxic content as well as on the balance of DA accumulation and depuration by shellfish (Mafra et al., 2010).

There are no reported cases of human illness associated with DA in any European countries or regions other than North America. However, in the absence of formal reporting systems, it cannot be assumed that mild cases have not occurred. Furthermore, the data relating to cases of human poisoning caused by DA are limited, except for a unique ASP outbreak in Canada in 1987. Such event involved 150 people with 19 hospitalization and 4 deaths after consumption of contaminated mussels (Jeffery et al., 2004; Álvarez et al., 2015).

Effective seafood monitoring programs for the detection of DA in the shellfish and coastal waters worldwide have been implemented by many regulatory agencies and therefore, human ASP events have not been documented since the first outbreak in 1987.

Domoic acid has also a toxic effect on marine wildlife and many poisoning events have been described in marine birds and mammals. A chronic DA epileptic syndrome was characterized in sea lions between 1998 and 2006. Therefore, sea lions are used as a sentinel species able to predicting potential hazard for human health (Lefebvre and Robertson, 2010).

## Diarrheic Shellfish Poisoning

This syndrome is one of the known intoxications caused by marine biotoxins and represents a frequent concern in shellfish industries, because it can cause a prolonged closure of mussel harvesting activity. The interruption of mussel sales and early public announcements are highly effective in controlling DSP outbreaks (Chen et al., 2013).

The accumulation of DSP toxins in any shellfish species is still little known. These toxins are produced by dinoflagellates belonging to the genera *Dinophysis* and *Prorocentrum*, even if the first genus is considered the main source, whereas *Prorocentrum* species are benthic and thus unavailable for suspension-feeding mussels (Nielsen et al., 2016).

Diarrheic shellfish poisoning toxins are polyether compounds with distinctive chemical structures grouped into four structural classes (Berti and Milandri, 2014): okadaic acid (OA) and its derivatives (dinophysistoxin or DTX); pectenotoxin (PTX); yessotoxin and its derivatives (YTX) and azaspiracid (AZA). The symptoms caused by OA group include diarrhea, nausea, vomiting, and abdominal pain, starting 30 min to a few hours after consumption of contaminated shellfish, with complete recovery within 3 days (Trainer et al., 2013). Among the other mentioned classes, PTX and YTX were first believed to be relevant in DSP syndrome due to their co-occurrence in shellfish with OA group, but they have not be implicated in human illness (Li et al., 2012), whereas AZA causes a form of poisoning characterized by nausea, vomiting, diarrhea, and stomach cramps.

Regarding to OA group toxins, it has been reported that they are soluble in fats and easily cross the cell membrane determining an inhibition of serine and threonine phospho-protein phosphatases (EFSA, 2008a).

Pectenotoxins are cyclic polyethers of marine origin and PTX-2 is the main toxin of such group. They have been associated with cases of diarrhea and other symptoms similar to those induced by OA and its derivatives (Poletti et al., 2003). They disrupt actin in the cytoskeleton, and may cause cell cycle arrest and cell death (Espiña et al., 2008).

Yessotoxin and its analogs are polyether toxins produced by dinoflagellates *Protoceratium reticulatum, Lingulodinium polyedrum*, and *Gonyaulax spinifera*. Even if they have been associated with DSP group, they do not cause diarrhea or inhibition of protein phosphatases (Paz et al., 2008) and their symptoms are still unknown in humans (Visciano et al., 2013).

Azaspiracids are nitrogen-containing polyether toxins comprising an unique spiral ring assembly containing a heterocyclic amine and an aliphatic carboxylic acid moiety. Even if 21 different analogs have been identified, AZA1, AZA2, and AZA3 are the most important ones depending on occurrence and toxicity (EFSA, 2008b).

Vale (2012) reported that the contamination of Portuguese shellfish with DSP toxins is an annually recurrent phenomenon, influenced by meteorological parameters affecting the blooming of the causative toxic microalgae, belonging to genus *Dinophysis*.

High levels of DSP toxins were also detected in shellfish harvested along the Chinese coast causing illness in more than 200 people in the year 2011 (Li et al., 2012). In the same year, 57 cases were described from Zheijang Province, China (Chen et al., 2013).

A DSP outbreak involving three people after the consumption of contaminated mussels was reported in Washington State (Trainer et al., 2013). Coincidentally 62 DSP illnesses occurred in British Columbia due to the ingestion of Pacific coast mussels, representing the first report of DSP in Western Canada (Taylor et al., 2013).

The DSP toxins are the most frequent and abundant marine biotoxins regularly found in shellfish from Southern European coastal areas (Braga et al., 2016). In France 11 DSP outbreaks involving 45 individuals were described in 2009. The contaminated mussels contained OA group toxin concentrations approximately eight times higher than what the European regulatory limits provide (Hossen et al., 2011). In 2010, more than 300 people in Northern Italy were poisoned by OA contaminated mussels (Bacchiocchi et al., 2015).

Symptoms of intoxication caused by YTX in humans are still unknown due to the fact that no human intoxication has been reported to date. However, toxicological studies carried out in rodents showed that YTX can be highly toxic when injected intraperitoneally (Visciano et al., 2013). Furthermore, PTX do not induce DSP-like symptoms even if acute toxicity can be observed in MBA (Wang et al., 2015). High levels of YTX were reported in shellfish farmed along the North Adriatic coast in 2004 correlated with the increasing presence of *G. spinifera* (Bacchiocchi et al., 2015).

Among the most recently identified groups of toxins causing human intoxication, AZA has been associated with shellfish poisoning in several people after consumption of mussels in the Netherlands in 1995. After that first event it was found in mussels harvested all over Europe, North-West Africa and Chile.

Some other outbreaks due to AZA were described in North-Western Ireland, affecting 12 people after the consumption of locally cultivated mussels; 10 individuals became ill with DSP symptoms in Ravenna (Italy) and 400 poisonings were reported in Belgium from mussels cultivated in Denmark (Furey et al., 2010).

## Neurotoxic Shellfish Poisoning

The brevetoxins (BTX) produced by the “Florida red tide” dinoflagellate *Karenia brevis* are polyether ladder compounds responsible for massive fish and marine mammal mortality above all in the Gulf of Mexico (Cassell et al., 2015) but also along the East coast of the United States and New Zealand (EFSA, 2010a). In humans, BTX are the causative agents of NSP and asthma-like symptoms through inhalation exposure (Sun et al., 2016).

Neurotoxic shellfish poisoning is characterized by both neurological and gastrointestinal effects which include nausea, vomiting, diarrhea, parasthesia, cramps, bronchoconstriction, paralysis, seizures, coma and, in extreme cases, may lead to death (Watkins et al., 2008).

On the basis of their molecular structures formed of 10–11 *trans*-fused rings, BTX have 2 skeletal backbones: A- and B-type and a variety of side chain substituents on the rings distal to the lactone (Cassell et al., 2015). They bind to and activate the voltage-gated sodium channels in cell membrane causing depolarization of neuronal and muscle cell membranes (EFSA, 2010a).

Brevetoxins have been implicated in the death of large numbers of fish and in morbidity and mortality of marine mammals (Gebhard et al., 2015).

There has been only a small number of sporadic cases of NSP in the United States in humans, with hospitalization but no fatalities. Outbreaks occurred in Florida with 2 cases in 1995, 3 in 1996, 2 in 2001, and 4 in 2005. In last event, 2 out of the 4 patients were children, more seriously affected than the adults. Another outbreak characterized by severity of symptoms was reported in 2006 in Florida (Watkins et al., 2008).

Respiratory effects associated with aerosolized red tide involve sneezing, throat irritation, burning, and itchy; in the case of asthmatics individuals a significantly increase of these symptoms has been observed (Fleming et al., 2007; Bean et al., 2011).

## Ciguatera Fish Poisoning

Ciguatera fish poisoning (CFP) is the most common foodborne illness worldwide with 50,000–500,000 incidences *per annum* (Mattei et al., 2014). The causative agents are toxins belonging to the CTX group (Hossen et al., 2015). Such syndrome results from the bioaccumulation and metabolism of precursor toxins along the fish food web. Precursor toxins (named gambiertoxins) are produced by benthic dinoflagellates of the genus *Gambierdiscus*, whose distribution includes tropical and subtropical coral reef areas, and accumulated by large predatory fishes, such as Spanish mackerels, moray eels, barracuda and snappers (Litaker et al., 2009; EFSA, 2010b; Chan, 2015).

Ciguatoxins are lipid-soluble polyether compounds consisting of 13–14 rings fused by ether linkages into a rigid ladder-like structure. More than 20 CTX analogs have been identified (EFSA, 2010b). At the cellular level, CTX activate the sodium ion channels causing cell membrane excitability and cell disruption (Hidalgo et al., 2002).

With regard to symptomatology, the acute period (24 h) is characterized by gastrointestinal problems (nausea, vomiting, abdominal pain, and diarrhea) whereas cardiovascular (bradycardia and hypertension) and neurological complications may occur within a few hours to 2 weeks after exposure, such as paresthesias, disesthesias, and hyperesthesias (Silva et al., 2015).

Even if the occurrence of CTX is generally restricted to some specific areas, in recent years a spread of ciguatoxic fish has been observed near European coasts and in the Mediterranean Sea. Recently, 6 outbreaks involving 28 people between 2010 and 2011 and one event with 20 cases in 2012 were described in New York and Northern Germany, respectively (Mattei et al., 2014).

A survey about the epidemiology of CFP in Asia reported 3 large outbreaks involving 100–200 patients in China, whereas 11 cases occurred between 1991 and 2008 in Taiwan. Among them, there was one death from ciguatera in 1998. In Malaysia 11 individuals were hospitalized in 2010 after the consumption of head and viscera of contaminated fish and 33 outbreaks affecting 103 subjects were described in Japan (Chan, 2015).

## Cyclic Imines

This group includes spirolides (SPX), gymnodimines (GYM), pinnatoxins (PnTX), and pteriatoxins (PtTX); SPX and GYM are produced by algal species from the genera *Alexandrium* and *Karenia*, whereas *Vulcanodinium rugosum* has been identified as the producer of PnTX (McCarthy et al., 2015). Recent studies suggest that PtTX are biotransformation products of PnTX in shellfish (Selwood et al., 2010). On the basis of their chemical structures, CI are macrocyclic compounds with imine and spiro-linked ether moieties (EFSA, 2010c). These moieties, common to all the members of CI, are thought to be the main structural determinants for their toxicity. Recent studies showed that CI antagonized both muscle type and heteromeric and homomeric neuronal nicotinic acetylcholine receptors as well as muscarinic acetylcholine receptors (Hellyer et al., 2013).

No acute symptoms have been recorded in humans and the toxicity of SPX and GYM is still being explored in mammalian models (Marrouchi et al., 2013); they induce a rapid death (within minutes) in laboratory mice injected intraperitoneally but they are often co-extracted with other lipophilic toxins such as OA and its analogs producing false positive in MBA test (Salgado et al., 2015). The first isolation of SPX was reported by Hu et al. (1995) from shellfish collected along the South Eastern coast of Nova Scotia, Canada, whereas GYM was isolated and characterized in the early 1990s from New Zealand oysters (MacKenzie, 1994).

Pinnatoxins and PtTX were originally identified in Japan in shellfish belonging to the genera *Pinna* and *Pteria*, respectively (Rundberget et al., 2011).

No acute poisoning events have been linked to contamination by PnTX and only one outbreak was reported associated with the bivalve of genus *Pinna*. However, PnTX show fast-acting toxicity when injected intraperitoneally into mice (Hess et al., 2013).

Despite the high acute toxicity in MBA, CI are not currently regulated in seafood due to the fact that acute poisoning in humans have not been directly related to shellfish contamination (Harju et al., 2016).

## Palytoxin

Palytoxin are potent non-protein marine compounds produced by corals belonging to the genus *Palythoa* and dinoflagellates belonging to the genus *Ostreopsis*. Several analogs have been identified (Biré et al., 2013).

Such group of toxins involves complex polyhydroxylated compounds with both lipophilic and hydrophilic areas.

Several symptoms were described after the consumption of shellfish and included a metallic taste, gastrointestinal malaise, diarrhea, nausea, vomiting, ataxias, dizziness myalgia, dyspnea, convulsion, and bradicardia.

About 200 people showed cutaneous and respiratory problems after exposure to marine aerosols containing PlTX in 2005 in Italy, whereas similar symptoms were reported between 2006 and 2009 in France (Biré et al., 2013). In literature, some cases of intoxication were fatal (Deeds and Schwartz, 2010; Aligizaki et al., 2011).

## The European Union Legislation

The EU food legislation focuses also on bivalve molluscs, giving space to them in a specific section in the Annexes to the Regulations of the “Hygiene Package”. In particular, Regulation (EC) No 853/2004 provides the maximum limits for marine biotoxins and Regulation (EC) No 854/2004 establishes monitoring and sampling plans in the production areas of live bivalve molluscs.

The monitoring of biotoxins in molluscs and health effects due to their consumption are important tasks for seafood control, because marine biotoxins may cause serious diseases in humans. According to the above mentioned Regulation 854/2004/EC, the production areas are periodically monitored to check the presence of toxins-producing plankton and their occurrence in live bivalve molluscs. Such monitoring is generally applied weekly during the periods when harvesting is allowed, but the sampling frequency must be representative for the considered area, taking into account the possible variations in the presence of plankton containing marine biotoxins. Such frequency may be reduced if the risk assessment on toxins or phytoplankton occurrence suggests a very low risk of intoxication. On the contrary, if the results of monitoring exceed the regulatory limits the production area shall be closed by the competent authority and can be re-opened when at least two consecutive results of biotoxin levels in molluscs meet with legislation. The closure of the production area is necessary in order to ensure that molluscs harmful to human health are not placed on the market. In fact the prevention of contaminated seafood reaching the markets is currently an effective way to protect human health (Berdalet et al., 2015).

Regulation (EC) No 2074/2005 highlights that the proposed maximum levels are based on provisional data and should be reassessed once new scientific evidence becomes available. The subsequent regulations amending the last one have provided internationally recognized methods for the different marine biotoxins. The increased complexity of biotoxin classes points out how the development of additional tools in the monitoring of production areas and molluscs is necessary. Testing methods for the detection of marine biotoxins can be implemented and refined as well. Actually these methods can be biological, functional or chemical assays, each of them showing advantages or disadvantages that must be taken into account.

The biological methods use animal models such as rats and/or mice. They are not very sensible and can be subject to interferences. Moreover, they do not give information about the concentration of the different toxins and it is difficult to identify which toxin causes the death of the mice. They are also time consuming and expensive. Even if they can have ethical problems, the MBA allows to detect new or unknown marine biotoxins during the periodic monitoring of production areas.

The functional methods use the capacity of marine biotoxins to trigger specific responses by interacting with a cellular component that selectively recognizes their structures and behaves as a receptor in sensitive systems, thereby transforming the chemical information of the incoming ligands into defined cellular effects. Therefore, the analytical challenge posed by the extreme complexity of toxin profiles is handled by the biological system, whose responses are specific for any toxin group. The main disadvantages are represented by the employment of viable cell strains, the potential of interferences and a particular expertise of laboratory technicians.

The chemical analytical methods are the current most powerful analytical tools able to identify multiple toxins. They are based on liquid chromatography (LC) to separate marine biotoxins by an extraction step, followed by the toxin-specific detection by UV (LC–UV), fluorescence (LC–FL), or mass spectrometry (LC–MS/MS). Due to the compound specific detection, the obtained results relate to individual concentrations of the investigated compounds and can be transformed into toxic equivalent, using conversion factors.

The MBA used for detecting PSP toxins is an official AOAC method, which has been used for more than 50 years in many Member States. Such method lays down that, initially one, but preferably 2 or 3 mice are injected intraperitoneally with 1 mL of an acid extract of molluscs. The time of death is recorded and if it lasts less than 5 min, dilutions of the extract are necessary, until the time of death becomes equal to 5–7 min. At that point three mice are injected and the average time of death is determined. The toxic concentrations are calculated in Mouse Units (MU) multiplied by the dilution factor and, if necessary, by a weight correcting factor, giving the result in MU/100 g. The conversion from MU into μg of STX equivalent is obtained multiplying by a conversion factor, which is calculated by each laboratory and periodically controlled.

The HPLC chemical method with fluorimetric detection and pre-column oxidation is applicable for the determination and quantification of most PSP toxin groups. Other chemical methods to determine PSP toxins, for instance LC–MS, are still being developed.

Other methods able to detect STX-group toxins are biomolecular methods, suitable however for *screening* purposes only. The biomolecular methodologies for toxins of the STX group are based on three different strategies by means of receptors, characteristics of cytotoxicity and antibodies. With regard to the latter approach, it is important to point out that, although the antibodies are very sensitive, their main problem to detect toxins of the STX group is the lack of good cross-reactivity to all the members of the group. As the differences of toxicity among the compounds of such group might be very high too, not always the toxicity corresponds to the toxin levels quantified by the antibody.

The official tests for the DA group toxins are based on the LC with UV detection. As a *screening* system for that group of toxins, it is possible to use also an LC–MS method that is completely intra-laboratory validated. The Regulation (EC) No 1244/2007 suggested as a *screening* procedure for toxins of the DA group the use of the 2006.02 ASP method based on the *Enzyme-Linked Immunosorbent Assay* (ELISA). It shows some advantages because it is sensitive and rapid, can be automated, requires a minimum training and does not require expansive equipment. However, it must be considered that in case of conflicting results, the HPLC chemical method with UV detector is the only valid reference method.

A Standard Operating Procedure was validated under the coordination of the European Reference Laboratory for marine biotoxins (EURL-MB) in an inter-laboratory validation study carried out by the National Reference Laboratories of Belgium, France, Germany, Ireland, Italy, Netherlands, Sweden, and the United Kingdom. Such method resulted highly specific and sensitive for the direct quantitative detection of all the four groups of regulated liposoluble toxins by the LC–MS/MS, using the certified reference materials for each compound to be investigated. The chromatographic separation of toxins is performed by gradient elution. Results are reported per toxin group as requested by the EU legislation and the total toxicity is calculated using the toxicity equivalence factors (TEF) recommended by CONTAM group of EFSA.

A series of procedures, differing in the portion (hepatopancreas or whole body) to be tested and in the solvents used for extraction and purification, may be carried out to detect marine toxins as referred to in Regulation (EC) No 2074/2005 and further amendments. In particular, two different analytic procedures have been optimized: for species of molluscs with a digestive gland of large dimensions (mussels, oysters, razor-shells, scallops, etc.), the procedure of hepatopancreas (20 g) must be used, whereas for species of molluscs where the digestive gland has small dimensions (clams, tellins, etc.) or is absent (tunicates, echinoderms, marine gastropods, etc.) the procedure of whole body (100 g) must be applied. The standard operating procedure provides a further protocol, whose application is recommended when the presence of YTX in the sample is suspected.

However, the lack of specificity of the biological test for DSP and its inadequacy for satisfying the regulatory limits prompted the ban of the MBA and its replacement with validated alternative methods. The Regulation (EU) No 15/2011 identified the LC–MS/MS as the reference method for liposoluble biotoxins, even if the MBA could be used as a *screening* method for new and unknown toxins.

## Author Contributions

MS and PV devised and drafted the review; MB, AM, and RT prepared the literature overview; GS contributed to manuscript revision.

## Conflict of Interest Statement

The authors declare that the research was conducted in the absence of any commercial or financial relationships that could be construed as a potential conflict of interest.
